# Supplementary material for: The Sharklogger Network—monitoring Cayman Islands shark populations through an innovative citizen science program
Source: PLoS One. 2025 May 9;20(5):e0319637. doi: 10.1371/journal.pone.0319637 (PMC12064031; doi:10.1371/journal.pone.0319637)
Supplement: S1 Fig — The package contains relevant information for the data collection. (PDF) [file pone.0319637.s001.pdf]

[redacted]

# The Sharklogger Network

Content:


1. The importance of logging dives with ZERO sharks
2. Anatomy of a shark
3. How to sex a shark
4. How to size a shark
5. Common shark species in Cayman and tips to avoid misidentifications
6. Data needed to identify individual sharks
7. Guide for data collection on shark behaviour
8. Guide for data collection on shark health


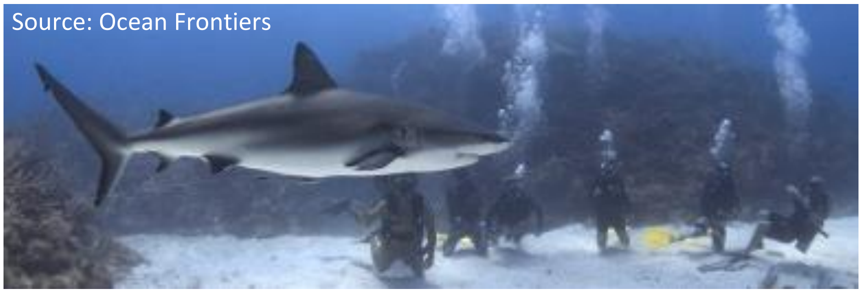


## 1. The importance of logging dives with ZERO sharks

*Why log ALL your dives and snorkels?*

One aim of monitoring our shark population is to find out where the sharks hang out in Cayman. By looking at the distribution pattern we can make assumptions on what might drive sharks to be abundant in certain areas and not so much in other areas. It also allows us to inform conservation management in case high shark abundance overlaps with high fishing activity, making the sharks more vulnerable in certain areas and/or times of the year.

Areas with a relatively high shark abundance will have more sharks per dive than areas where sharks are less likely to be seen. However, if we do not know how many times you did go diving at a certain area, we cannot calculate the "number of sharks per dive" at this area. Therefore, for this statistical analysis, it is necessary to count the number of dives with zero sharks.

Sighting reports, i.e. only the dives on which you have seen a shark, give us only the information of the date, location, time and depth of where a shark has been seen, it doesn't count the number of dives with zero sharks.

The report of all dives that you do, i.e. the dives on which you have seen a shark AND the dives on which you didn't see a shark, gives us the information on how big the effort was to see a shark. It gives us the count of the number of dives with zero sharks.

***What's the difference?***

For example, in June a diver went 30 times diving at Macabuca and saw one shark on 10 dives, totalling 10 sharks at Macabuca in that month.

The same diver also went diving 10 times from a boat at Tarpon Alley (North Wall) and saw one shark on each dive, totalling 10 sharks at Tarpon Alley in that month.

Check out how the number of sharks per dive differs depending on whether the diver reports ALL dives or dives with shark sightings only:

| | Total number of sharks seen | Total number of dives | Sharks per dive |
|---|---|---|---|
| | | **Macabuca** | |
| **Sightings only** | 10 | 10 | 1 shark per dive |
| **All dives** | 10 | 30 | **0.33 sharks per dive** |
| | | **Tarpon Alley** | |
| **Sightings only** | 10 | 10 | 1 shark per dive |
| **All dives** | 10 | 10 | **1 shark per dive** |

Clearly, we know that it is less likely to see a shark at Macabuca (0.33 sharks per dive) than at Tarpon Alley (1 shark per dive) and the analysis confirms this when we KNOW the effort. However, if we only have the sighting information, the analysis would show that there are equally many sharks at both dive sites (1 shark per dive).

Therefore, it is important to you report all the dives that you do, regardless of whether you have seen a shark or not, because without these "zero- shark" dives the analysis doesn't work and any potential trends cannot be confirmed nor denied.

2. Anatomy of a shark

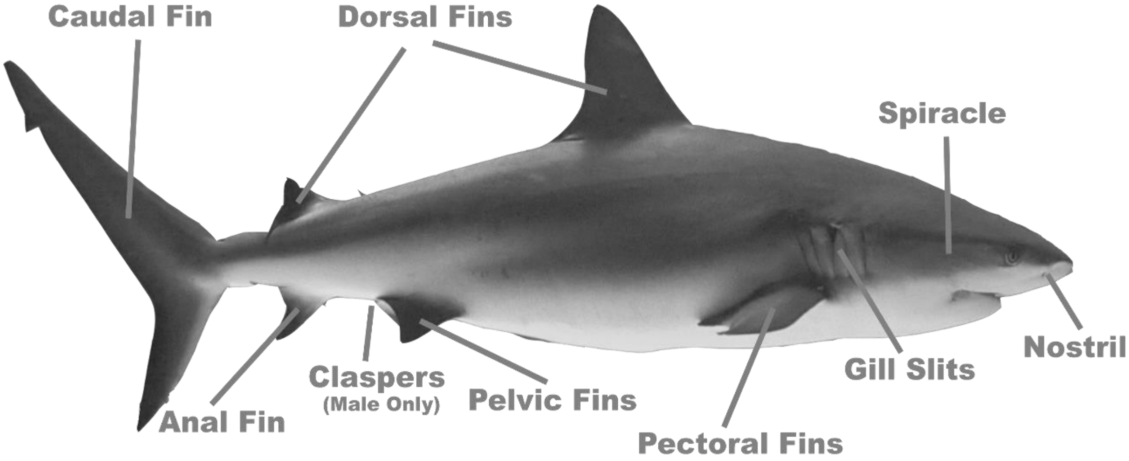


3. How to sex a shark

The sex of a shark can be identified by the presence or absence of claspers. Look at the underside of the shark at the pelvic fins. In males, a clasper will extend out from the inside edge of each pelvic fin. Females do not have claspers.

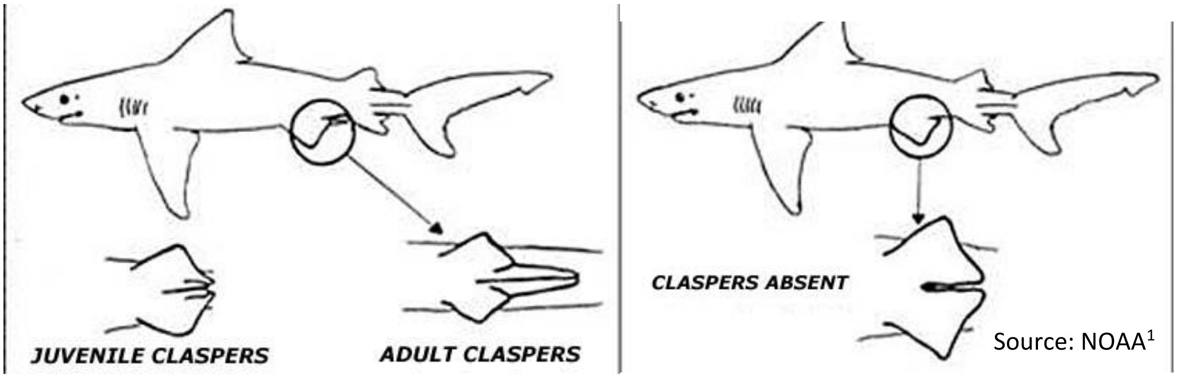


Source: NOAA[1]

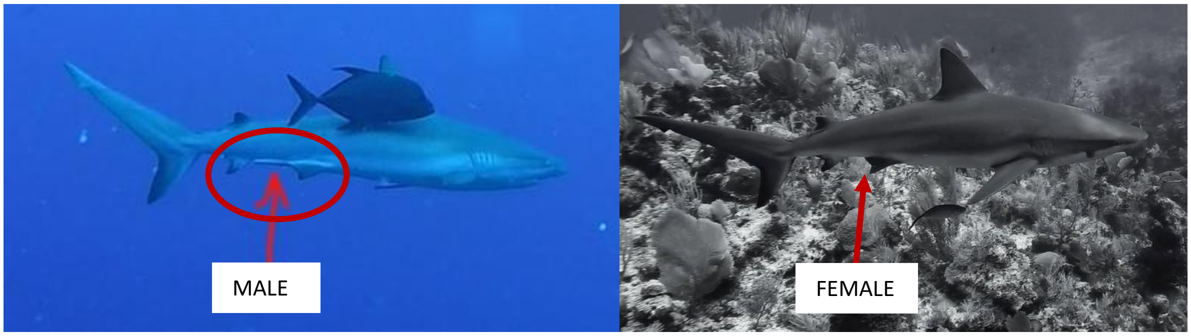


Source: Johanna Kohler, DoE

***Mature vs immature sharks***

Be careful when determining the sex of juvenile sharks (e.g. Caribbean reef sharks < 5ft and nurse sharks < 6ft). In juvenile males, the claspers might be smaller than the pelvic fins and not visible from the side (see comparison in picture below). If you see a (juvenile) shark but are unable to inspect the pelvic region close up or are unsure whether the shark has claspers or not, please state “**unknown**” instead of “female or male”.

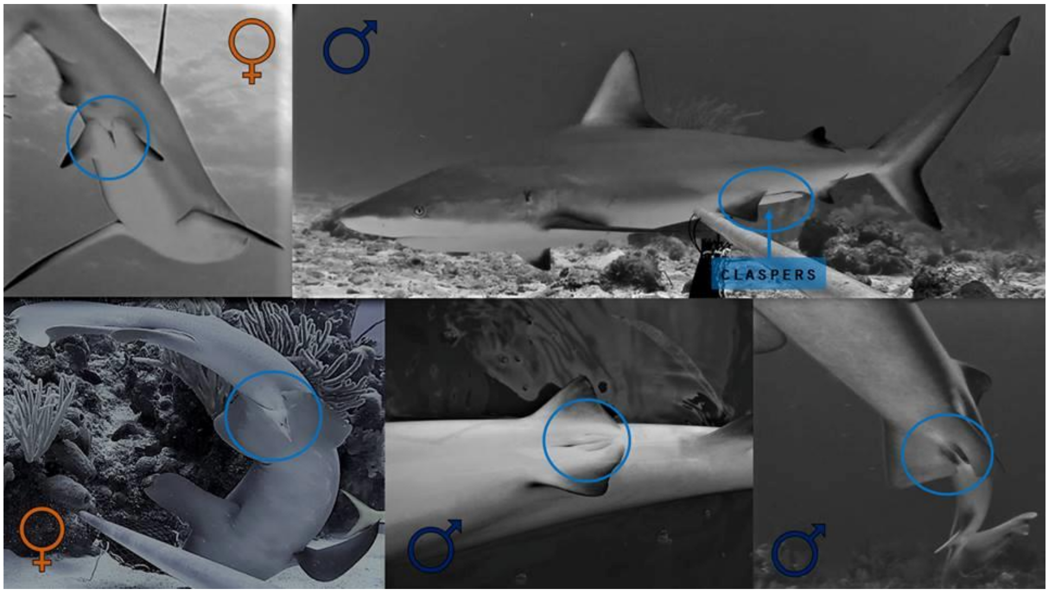


Source: Johanna Kohler, DoE

4. <u>How to size a shark</u>

Estimate the size of the shark from the tip of the snout to the tip of the tail. This is called total length. Report it in your preferred unit (ft or m) and estimate it to the nearest 10 cm/ inch. Use objects under water as reference to help you estimate the size. For example, compare the size of the shark to that of other divers, the reef, corals, the boat, and the boat ladder.

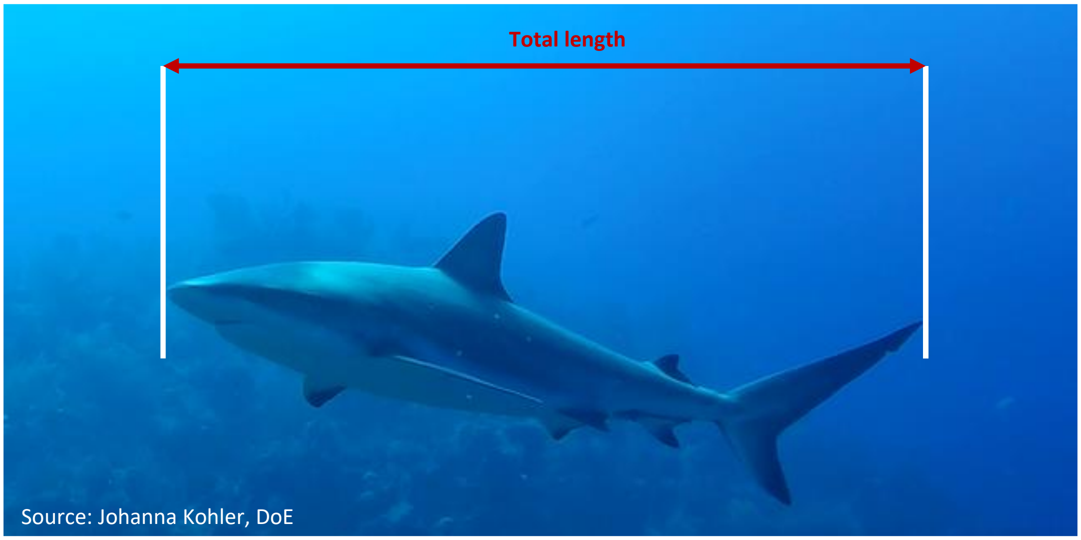


Source: Johanna Kohler, DoE

5. Common shark species in Cayman and tips to avoid misidentifications

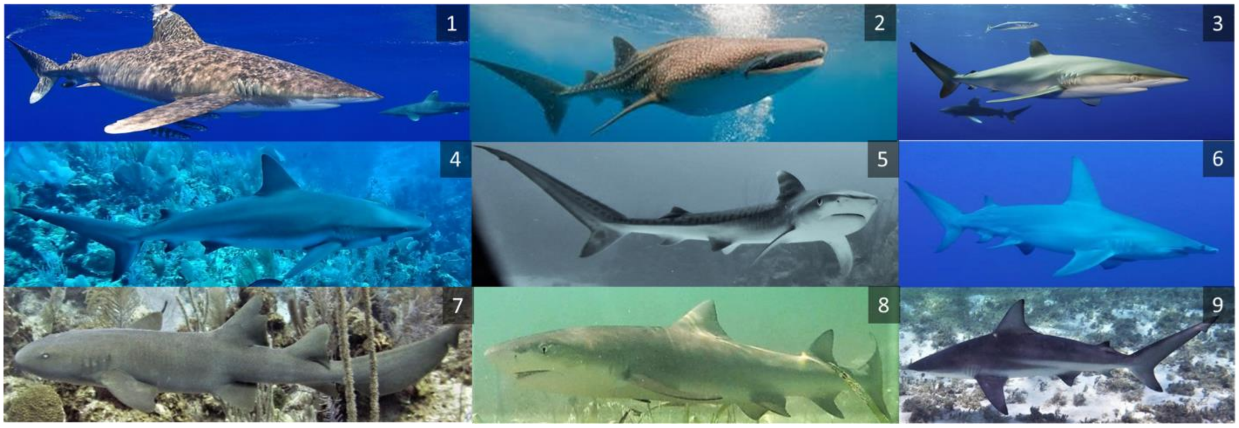


1. Oceanic whitetip shark, 2. Whale shark, 3. Silky shark, 4. Caribbean reef shark, 5. Tiger shark, 6. Great hammerhead, 7. Nurse shark, 8. Lemon shark, 9. Blacktip shark.
Source: Johanna Kohler, DoE

1.-3.: these species are mainly oceanic and therefore less likely to be encountered on the reef. 4.-9.: these species are coastal sharks and therefore more likely to be encountered on the reef. Highly reef-associated species such as 4., 7., 8., and 9. are more common than 5. And 6. Which might be seen at depth and/or out in the blue off the drop off.

*How to differentiate Caribbean reef and blacktip sharks?*

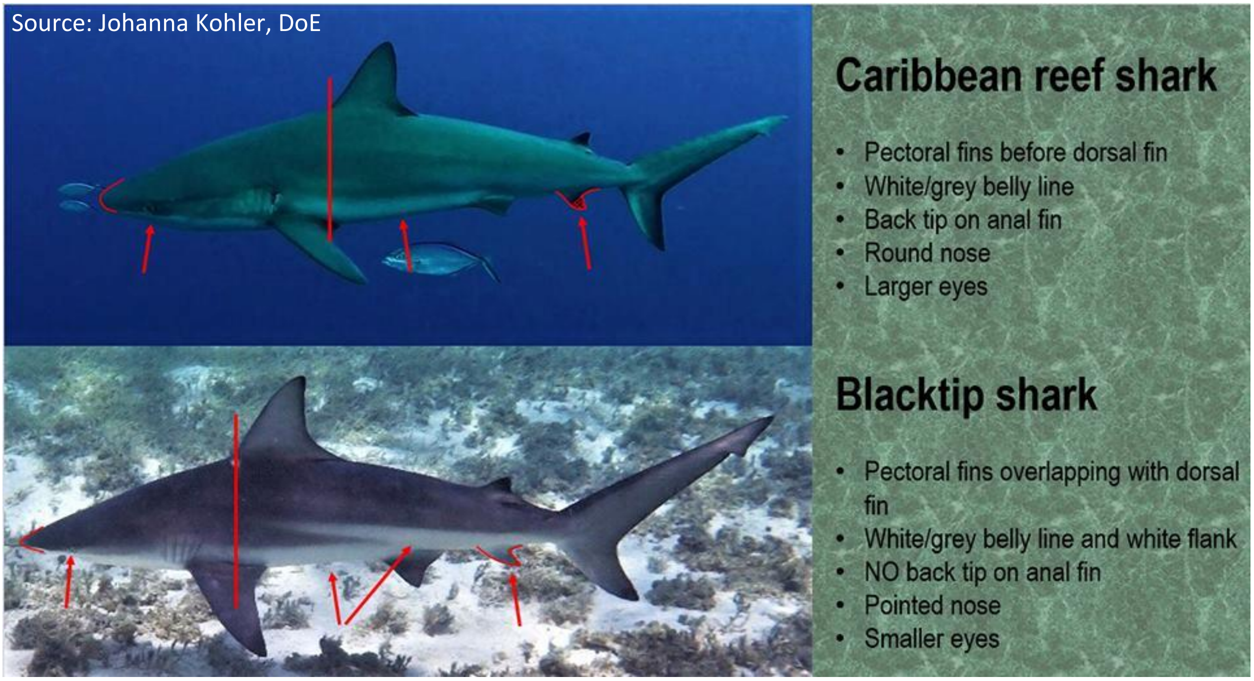

Source: Johanna Kohler, DoE

## 6. Data needed to identify individual sharks

Let us know if a shark is tagged, you know the shark, or if it has any distinguishing markings that could help for subsequent identification. This way we can track individuals over time and estimate the size of their home range.

*What to look out for*

*1. Tagged sharks – look out for dorsal fin tags*

The DoE research team has been tagging sharks since 2009. Sharks are marked with an orange dorsal fin tag. However, when at depth the colour might look different (less bright orange but faded) as orange is one of the colours that is filtered out by the water within the first few meters/feet. Also, sharks that were tagged in previous years may have lost their tag or the tag has overgrown with algae. So if you see a shark, please have a second look whether the dorsal fin has one of the following markings:

- orange tag (if you can, report number on tag, e.g. DoE 116)

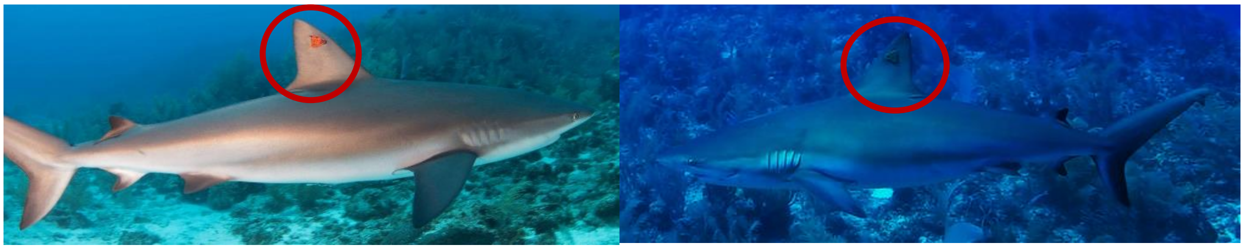

- green, algae overgrown tag

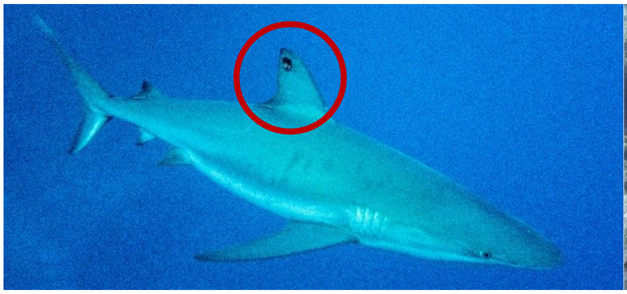

- hole or dark spot (scar) from lost tag

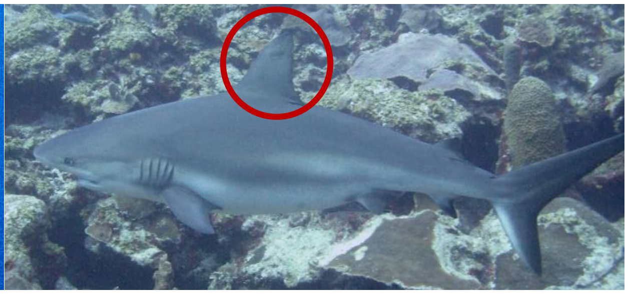

Source: Johanna Kohler, DoE

*2. Frequently encountered residents*

Some sharks, that are frequently encountered by divers in certain areas and are recognizable through distinct features, have names such as the two examples below. If you do see a shark of which you know the name, report the name together with its distinguishing markings.

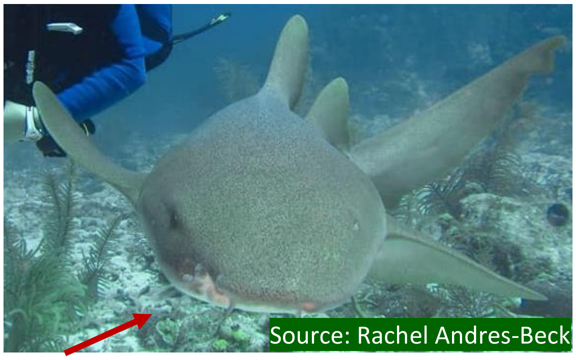

Source: Rachel Andres-Beck

<u>Example 1:</u> Nurse shark named Finn
aka Amigo aka Kiki.

*Distinguishing feature*: scar/deformation on the right corner of his mouth

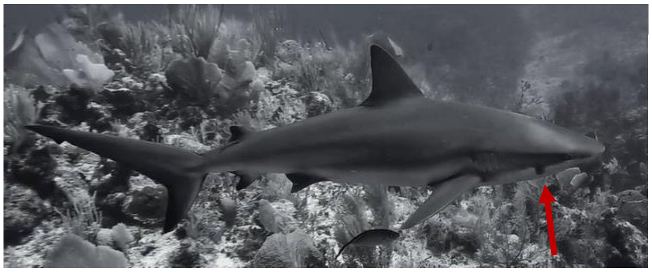

Source: Johanna Kohler, DoE

<u>Example 2:</u> Caribbean reef shark named
Scarlet aka Smudge

*Distinguishing feature*: black birth mark on the right cheek

### *3. Distinguishing features*

Report any hooks, scars, skin discolorations, dorsal fin shape (e.g. nicks) in the comment section of your Shark Log. This allows us to identify individual sharks and track their movements over time, even if an individual is unknown to divers (and doesn't have a name).

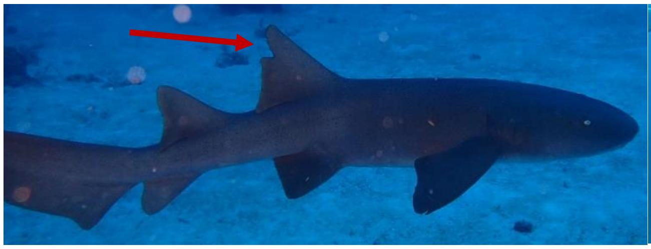

Nurse shark
Sex: unknown
Markings: Large nick in first dorsal fin

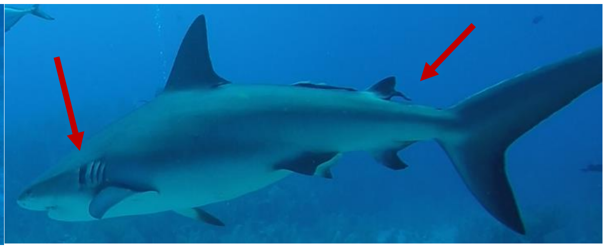

Caribbean reef shark
Sex: female
Markings: damaged gill on left side, 2nd dorsal fin it deformed

Nurse shark
Sex: unknown
Markings: skin discolouration (black band) on left side of tail, between 1st and 2nd dorsal fin

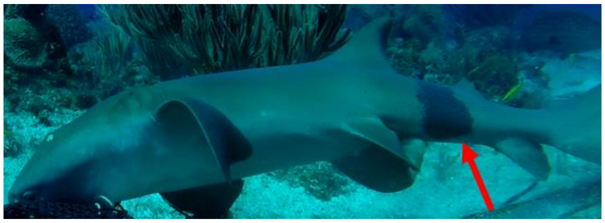

Source: Johanna Kohler, DoE

7. Data collection on shark behaviour

Please report any "unusual" behaviour and behaviour that you find interesting. These can include interactions of sharks with divers, other fish, and other sharks.

Please also report if sharks "hunt" for lionfish on their own.

Please report mating events and any behaviour or encounters related to reproduction. See details below to identify each behaviour:

- *Mating event*

Sharks reproduce via internal fertilisation. Male sharks have two claspers, extensions of their pelvic fins, used to transfer sperm. The male bites the female just behind the pectoral fin and pins her head against the substrate to then insert only one clasper. Once inserted, claspers are equipped with spurs to stay in place and the male expels sperm into the female along with seawater. The eggs are fertilized inside the female's body.

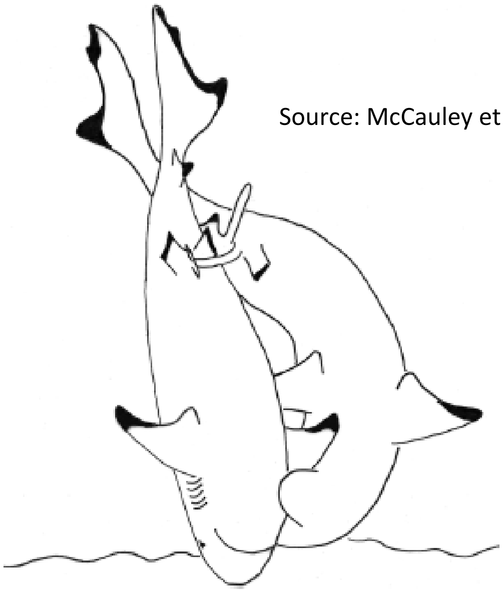

Source: McCauley et al., 2010

- *Mating behaviour e.g. courting*

During mating season, you might observe the following behaviour depending on the species:

Caribbean reef shark: Swimming (relatively fast) along the wall in one direction, tailing each other (usually female followed by a male). Male will try to catch the female in order to bite the pec/side of the female (as mentioned above). Female might try to escape, leading to change of direction with the male following her again.

Nurse shark: Might be found in groups, resting together, circling each other, "chasing the tail" of the other shark(s).

- *Mating signs ("love bites") on female sharks*

As the male will bite the female during mating, females will receive bite wounds during the event. Because of this, female do have thicker skin than males and the relatively fast self-healing of sharks helps to withstand the males biting and with recovery after.

The picture below shows a female with fresh mating wounds (red, on left) and a few weeks later with almost healed scars (white, on right)

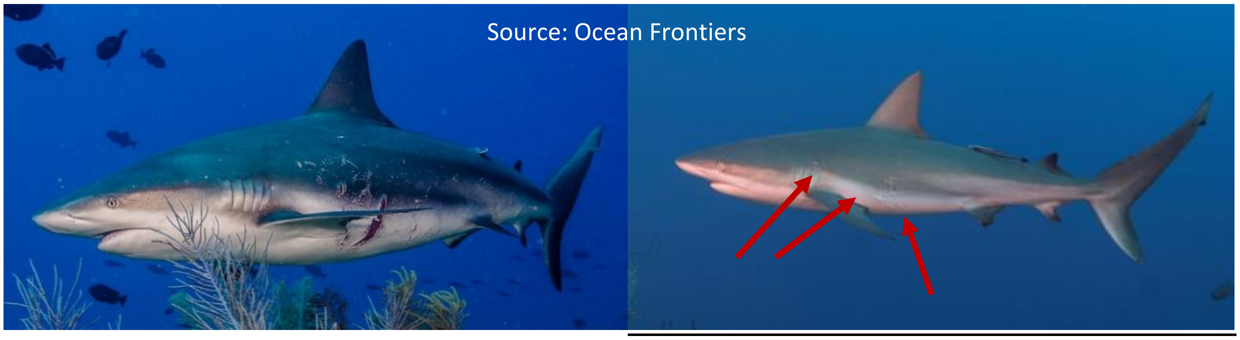


## 8. Data collection on shark health

Report all injuries, hooks, fishing lines, skin disease (discolouration, bumps, growths), and parasites (not remoras).

Example 1: Caribbean reef shark, hook in right corner of mouth

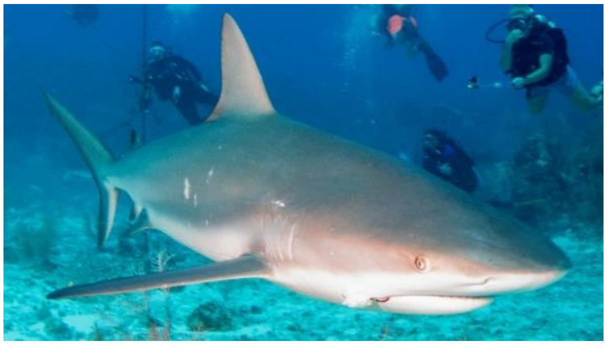

Example 2: nurse shark, bit or cut on tip of nose, large chunk missing

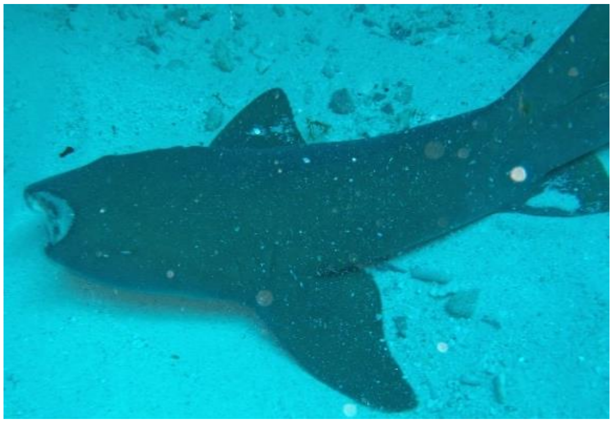

**Taking your underwater camera on your dives?**

If you happen to take pictures or videos of sharks that's great!

Please submit the footage that you would like to share alongside your Shark Log of that particular month. The footage will be added to your Shark Log and the DoE database to aid identification of individuals and other research aims (e.g. documenting behaviour of interest or monitoring health of individuals).

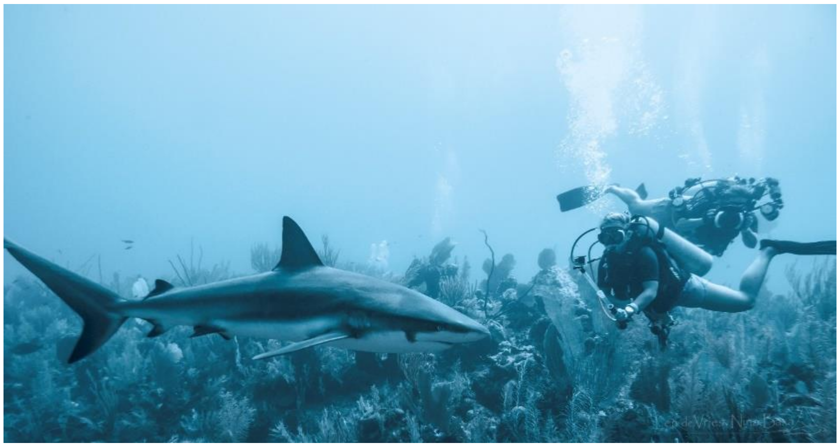

**THANK YOU** for your help with the monitoring of Cayman's sharks!

If you have any questions or concerns, please get in touch.

---

# Contact


Email: [redacted]

Johanna Kohler, DoE shark project officer: [redacted]

DoE office: [redacted] / www.doe.ky

DoE Enforcement
Grand Cayman: [redacted]
Little Cayman: [redacted]
Cayman Brac: [redacted]


---

**Resources**


https://www.fisheries.noaa.gov/new-england-mid-atlantic/atlantic-highly-migratory-species/tagging-instructions-and-resources-volunteers

McCauley, D.J., Papastamatiou, Y.P., Young, H.S., 2010. An Observation of Mating in Free-Ranging Blacktip Reef Sharks, Carcbarbinus melanopterus. Pacific Sci. 64, 349–352. https://doi.org/10.2984/64.2.349

Example of data entry in your Shark Log. Note the dives with “0” sharks:

| DoE Shark Project - Sharklogger Network | please email to: **sharkloggers@gmail.com** | [redacted] |
|---|---|---|
| **OBSERVER NAME** | **Name / Dive operator** | |
| **OBSERVER EMAIL** | email contact | |
| OBSERVER PROFESSION & / AFFILIATION | Private diver / dive staff / dive centre | |
| CITY OR DISTRICT & COUNTRY OF RESIDENCE | Grand Cayman / Little Cayman / Cayman Brac | |
| EVEL of DIVING & MARINE IDENTIFICATION EXPERIENCE (C=Considerable / M=Moderate / l | C / M / L | |

| **DATE** (DD/MM/YYYY) | **DISTRICT or PART OF ISLAND** (Name/N/NE/E etc.) | **DIVE SITE** (if has established name) | **TIME OF DAY** (approx.) | **DIVE TIME** (approx.) | **MAX. DEPTH** (approx.) | **TEMPERATURE** (Deg C) | **while Lionfishing?** | **VISIBILITY** (to nearest 5m/15ft) | **CURRENT** (weak 0 - strong 3) | **NO. of SHARKS SEEN** (state if ZERO!) | **SHARK SPECIES** (if known) e.g. Caribbean reef, blacktip, nurse shark | **SIZE** (approx. to nearest 10cm/4inch) | **Sex** (female / male / unknown) | **TAGGED / NAME / MARKINGS?** (Tag no. / colour / scars / hooks) | **No. of Divers / Guests** (if applicable) | **Comments** |
|---|---|---|---|---|---|---|---|---|---|---|---|---|---|---|---|---|
| 01-Apr-16 | West Side | Darvin's Theory | 10:00 | 55 | 90 | 80 | no | 80 | 0 | 0 | | | | | 3 | |
| 01-Apr-16 | West Side | Round Rock | 11:30 | 45 | 80 | 80 | no | 100 | 0 | 1 | Nurse shark | 4ft | female | no tag, hook in left corner of mouth | 15 | followed us the entire dive |
| 01-Apr-16 | West Side | Devil's Grotto | 13:30 | 50 | 40 | 79 | yes | 80 | 2 | 0 | | | | | 15 | |
| 02-Apr-16 | West Side | Meridian Drop Off | 09:30 | 50 | 90 | 79 | no | 90 | 0 | 0 | | | | | 8 | |
| 02-Apr-16 | West Side | Lone Star Ledges | 11:30 | 50 | 50 | 80 | no | 90 | 0 | 0 | | | | | 8 | |
| 02-Apr-16 | North Wall | Lemon wall | 02:30 | 45 | 50 | 80 | no | 100 | 0 | 0 | | | | | 3 | |
| 02-Apr-16 | North Wall | Eagleray Pass | 10:00 | 50 | 80 | 80 | no | 100 | 1 | 0 | | | | | 3 | |
| 02-Apr-16 | North Wall | Durgon's Domain | 10:00 | 45 | 80 | 80 | yes | 80 | 0 | 0 | | | | | 12 | |
| 02-Apr-16 | North Wall | Lemon reef | 12:30 | 45 | 40 | 80 | no | 80 | 0 | 2 | Nurse shark | 4ft + 7ft | unknown + female | no tags | 12 | small shark was sleeping under reef ledge |
| 03-Apr-16 | West Side | Round Rock | 10:00 | 45 | 80 | 80 | no | 80 | 0 | 0 | | | | | 14 | |
| 03-Apr-16 | West Side | Eagle's Nest | 10:30 | 45 | 80 | 80 | no | 60 | 0 | 0 | | | | | 15 | |
| 04-Apr-16 | South | Black Forest | 09:30 | 45 | 80 | 81 | no | 90 | 0 | 0 | | | | | 20 | |
| 04-Apr-16 | South | Laura's Reef | 11:30 | 50 | 50 | 80 | no | 80 | 2 | 0 | | | | | 20 | |
| 04-Apr-16 | West Side | Disneyworld | 14:30 | 50 | 100 | 81 | no | 90 | 0 | 1 | Caribbean reef shark | 6ft | male | yes, didn't see tag number | 3 | shy and cautious of divers |
| 04-Apr-16 | West Side | Kittiwake | 10:00 | 45 | 80 | 80 | no | 90 | 0 | 0 | | | | | 14 | |
| 04-Apr-16 | West Side | O.V. Wreck | 11:30 | 50 | 50 | 81 | no | 90 | 0 | 1 | Nurse shark | 7ft | male | Finn (hook scar on mouth) | 14 | very friendly |
| 04-Apr-16 | West Side | Darvin's Theory | 12:30 | 45 | 80 | 80 | no | 90 | 0 | 0 | | | | | 17 | |
| 04-Apr-16 | West Side | O.V. Wreck | 14:00 | 50 | 50 | 80 | no | 90 | 0 | 0 | | | | | 17 | |
| 05-Apr-16 | West Side | O.V. Wall | 09:45 | 45 | 80 | 80 | no | 80 | 0 | 0 | | | | | 9 | |
| 05-Apr-16 | North Wall | Tarpon Alley | 11:00 | 50 | 50 | 80 | no | 80 | 0 | 0 | Caribbean reef shark | 7ft | female | scar on dorsal from tag | 9 | Name "Spot" |
| 05-Apr-16 | North Wall | Creole Cliff | 14:30 | 60 | 50 | 80 | no | 70 | 1 | 0 | | | | | 2 | |
| 06-Apr-16 | West Side | Eagle Ray Rock | 12:30 | 45 | 80 | 80 | no | 70 | 0 | 0 | | | | | 10 | |
| 06-Apr-16 | West Side | Pallas Arches | 14:30 | 50 | 60 | 80 | no | 80 | 0 | 0 | | | | | 10 | |
| 06-Apr-16 | West Side | Big Tunnels | 12:00 | 45 | 100 | 79 | no | 100 | 0 | 0 | | | | | 2 | |
| 06-Apr-16 | West Side | Fish Reef | 14:00 | 50 | 55 | 79 | no | 70 | 2 | 0 | | | | | 2 | |
| 07-Apr-16 | West Side | Stewies Crack | 02:30 | 50 | 50 | 80 | yes | 80 | 0 | 0 | | | | | 4 | |
| 07-Apr-16 | North Wall | Ghost Mtn | 10:30 | 45 | 100 | 80 | no | 70 | 0 | 0 | | | | | 5 | |
| 07-Apr-16 | West Side | Doc Polson | 12:15 | 50 | 50 | 80 | no | 80 | 0 | 0 | | | | | 5 | |
| 08-Apr-16 | West Side | Alexs Alley | 12:00 | 45 | 100 | 80 | no | 80 | 1 | 0 | | | | | 3 | |
| 08-Apr-16 | West Side | Funky Sponge | 13:45 | 50 | 50 | 80 | no | 100 | 1 | 1 | Nurse shark | 4ft | unknown | scar on left hand side on head | 3 | |
| 09-Apr-16 | West Side | Meridian Drop Off | 09:30 | 45 | 80 | 80 | no | 100 | 0 | 0 | | | | | 10 | |
| 09-Apr-16 | North Wall | Roundabout | 11:00 | 50 | 50 | 80 | yes | 70 | 0 | 0 | | | | | 10 | |
| 09-Apr-16 | North Wall | Eagle Ray Pass | 14:30 | 50 | 60 | 80 | no | 70 | 1 | 0 | | | | | 4 | |
