# Supplementary figures and images for: The Sharklogger Network—monitoring Cayman Islands shark populations through an innovative citizen science program
Source: PLoS One. 2025 May 9;20(5):e0319637. doi: 10.1371/journal.pone.0319637 (PMC12064031; doi:10.1371/journal.pone.0319637)

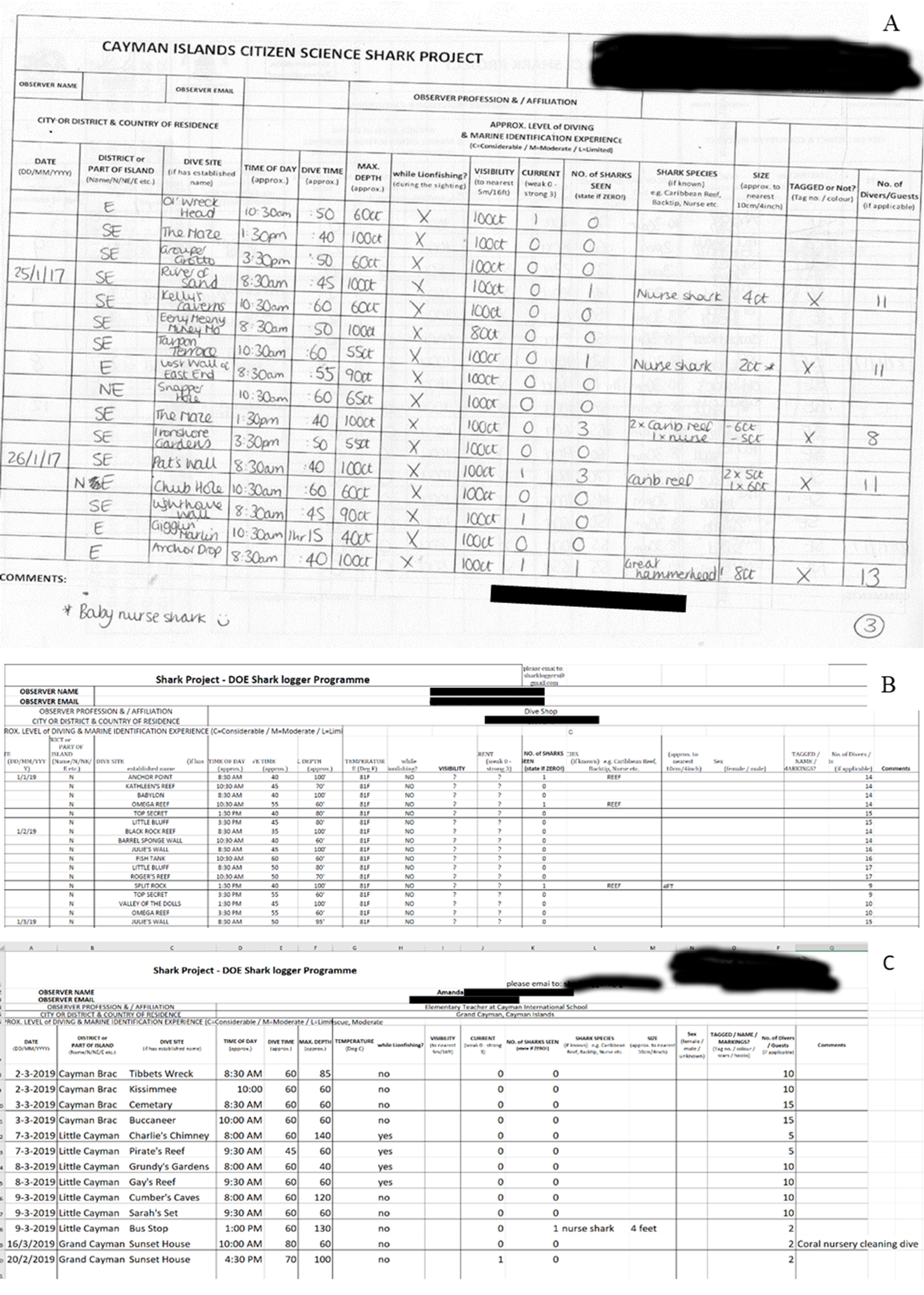

Supplement: S2 Fig — Personal information has been redacted. (TIF) [file pone.0319637.s002.tif]

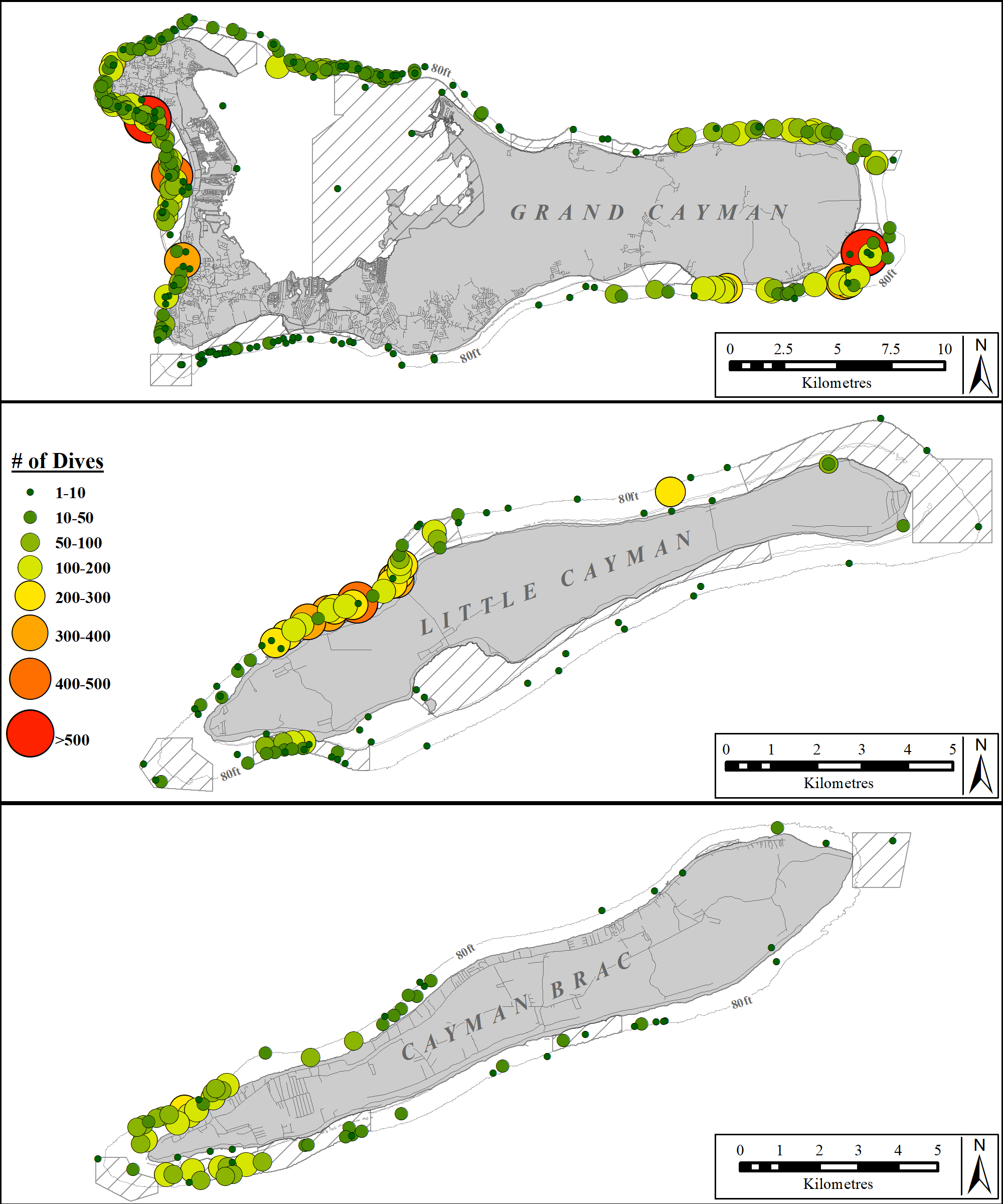

Supplement: S3 Fig — Circles indicate the location of dive sites accessible by boat or from shore, the circle size is scaled by the diving effort (number of dives). Dive sites with a greater number of dives are indicated by larger circles than dive sites with fewer dives. The color of circles and corresponding range of diving effort is shown on map. The line around each island indicates the 25m (80ft) depth contour and the shaded areas indicate the extent of MPAs. Created by the Department of Environment, Cayman Islands Government. Insert layer’s geography was developed by Esri and sourced from Garmin International, Inc., the U.S. Central Intelligence Agency (The World Factbook), and the National Geographic Society for use as a world basemap [82]. (TIF) [file pone.0319637.s003.tif]
