# Supplementary material for: The Sharklogger Network—monitoring Cayman Islands shark populations through an innovative citizen science program
Source: PLoS One. 2025 May 9;20(5):e0319637. doi: 10.1371/journal.pone.0319637 (PMC12064031; doi:10.1371/journal.pone.0319637)
Supplement: S1 Table — (PDF) [file pone.0319637.s004.pdf]

Variable	Sample protocol
time of day	The time of day was reported as, or as close as possible to, the actual time (e.g. 10:36) of the dive start.
dive location	The dive location was reported as the dive site name (in case of public moorings and common shore access points), description of site (in case of anchor drop and unusual shore dives), or coordinates (latitude, longitude).
dive duration	The dive duration was defined as the minute count (e.g. 56 min) from dive start to dive end. Most professional divers (i.e. dive operations) reported standard dive times (e.g. 60 min) of their dive centre, even if divers were slightly shorter or longer in the water.
maximum depth	The depth was defined as the diver's maximum depth (e.g. 26 m/85 ft) during the dive, the depth of snorkels was reported as 0 m, and professionals reported the standard max. dive depth (e.g. 80 ft/24 m) of the dive, regardless of individual depths.
water temperature	The temperature was defined as the minimum ambient temperature logged by the dive computer (the temperature automatically shown on instruments and most likely the temperature recorded at max. depth).
visibility	The visibility was estimated to the nearest 5 m/15 ft.
dive group size	Dive group size, the count of divers on a dive, was reported. In case of a boat dive, the dive group size was either the number of divers on the boat (mostly reported by professional divers) or of a subgroup (mostly reported by individual divers).
number of sharks per species	For each species, divers reported the shark count, the maximum number of individuals, encountered during the dive.
shark size	The shark size (total length, TL), defined as the distance from tip of snout to tip of tail, was reported as an estimate to the nearest 0.1 m/0.3 ft, using various references (e.g. other divers, objects) and personal experience.
sex of shark	The sex of individuals was determined visually through the presence (male) or absence (female) of claspers. Was the determination of sex not possible (e.g. the shark was too far away or resting on the bottom), the individual's sex was classified as 'unknown'. For the sex determination of immature (relatively small) sharks, divers were instructed to take caution and, if in doubt, report 'unknown' rather than a specific sex because claspers, although present, may be small and not seen by the diver.
dorsal fin tag	Report of whether a shark was tagged (yes/no) through the presence/absence or remains (e.g. scar) of the dorsal fin tag. In case of a tagged shark divers were instructed to record the number and/or condition of the tag (e.g. algae overgrown, lost tag) which may help subsequent identification of tagged individuals.
