# Supplementary material for: The Sharklogger Network—monitoring Cayman Islands shark populations through an innovative citizen science program
Source: PLoS One. 2025 May 9;20(5):e0319637. doi: 10.1371/journal.pone.0319637 (PMC12064031; doi:10.1371/journal.pone.0319637)
Supplement: S2 Table — (PDF) [file pone.0319637.s005.pdf]

Shark ID	Sex	Maturity	Name	Distinguishing markings
C107	F	M	107	algae overgrown / ‘degraded black’ dorsal fin tag (#107), very big and girthy
C137	F	M	no	scar on RHS pec fin/line on 1st dorsal fin
C143	F	M	no	skin overgrown dorsal fin tag (#143), bend over dorsal fin
C2	M	M	Bash Brothers, Gonzales	one shark has a scar on its LHS gills, two male sharks together
C3	F	M	Big Bertha	scar on RHS gill, deformed 2nd dorsal fin
C4	M	M	Twitchy, Scrub	black dot on tip of dorsal fin
C5	NA	IM	Little Basher	very shiny, small size, black mark on flank
C6	F	M	Spot, Shadow tag	scar from dorsal fin tag
C7	F	M	Scarlet	diagonal scar on left 3-4 gills, scar on LHS mouth
C8	F	M	Smudge, Scarlet	dark birth mark on RHS mouth
C9	F	M	no	new-ish tag, not overgrown
N1	M	M	Finn, Amigo, Kiki	hook scar on RHS of mouth
N10	M	M	Huckleberry, Huck	scar on bottom RHS jaw, 4 inch long
N11	NA	NA	Cuddles	nick in both dorsal fins
N12	F	IM	no	cuts on dorsal fin, small size
N2	M	IM	Henry, Gappy, Fernando, Princess Hook, Skip, Sidney, Hook, Little Hookie, Juju	large nick in 1st dorsal fin, hook (now scar) through one nostril and top jaw
N20	F	IM	no	very light in colour, nick in pec fin
N21	NA	IM	no	nick in caudal fin, usually inside wreck sleeping
N22	NA	IM	Fishy	very small (baby) shark, usually sleeps in crevice
N23	NA	M	no	very dark in colour, small scar on head, small size
N3	F	IM	Bella, Hero	multiple scratches on top of head, 2 light spots on LHS next to dorsal fin, 1 small spot on dorsal fin
N4	F	IM	no	algae overgrown dorsal fin tag
N5	NA	IM	Gashy	gash in head and through eye
N6	NA	IM	Gill	injured gill
N7	F	IM	no	hook and line in RHS mouth
N8	NA	IM	Tatty	tatty dorsal tip
N9	F	IM	Loopy	very light in colour, hook scar LHS mouth
