# Supplementary material for: The Sharklogger Network—monitoring Cayman Islands shark populations through an innovative citizen science program
Source: PLoS One. 2025 May 9;20(5):e0319637. doi: 10.1371/journal.pone.0319637 (PMC12064031; doi:10.1371/journal.pone.0319637)
Supplement: S3 Table — (PDF) [file pone.0319637.s006.pdf]

Variable	Number of shark logs	% of total shark logs
Total	24,442	100
Max depth	24,439	99.9
Current strength	22,703	93
Temperature	20,655	84.5
Dive duration	24,426	99.9
Time of day	24,340	99.6
Lionfish culling	24,442	100
Visibility	18,824	77
Dive group size	17,116	70
