# Supplementary material for: The Sharklogger Network—monitoring Cayman Islands shark populations through an innovative citizen science program
Source: PLoS One. 2025 May 9;20(5):e0319637. doi: 10.1371/journal.pone.0319637 (PMC12064031; doi:10.1371/journal.pone.0319637)
Supplement: S4 Table — Test statistic (Z) and p-values are reported and significant differences, at the 0.05 level, are marked with * . (PDF) [file pone.0319637.s007.pdf]

Species	Test statistic	silky shark	blacktip shark	Caribbean reef shark	nurse shark	tiger shark	lemon shark	whale shark
**blacktip shark**	Z	-0.613						
	p	0.270						
**Caribbean reef shark**	Z	-52.074	-51.461					
	p	**< 0.001***	**< 0.001***					
**nurse shark**	Z	-62.600	-61.987	-10.525				
	p	**< 0.001***	**< 0.001***	**< 0.001***				
**tiger shark**	Z	-0.136	0.477	51.939	62.464			
	p	0.446	0.317	**< 0.001***	**< 0.001***			
**lemon shark**	Z	-0.101	0.511	51.973	62.498	0.034		
	p	0.460	0.305	**< 0.001***	**< 0.001***	0.486		
**whale shark**	Z	0.001	0.614	52.075	62.600	0.136	0.102	
	p	0.500	0.270	**< 0.001***	**< 0.001***	0.446	0.459	
**hammerhead spp.**	Z	-3.470	-2.857	48.605	59.130	-3.334	-3.368	-3.470
	p	**< 0.001***	**0.002***	**< 0.001***	**< 0.001***	**< 0.001***	**< 0.001***	**< 0.001***
