# Supplementary material for: The Sharklogger Network—monitoring Cayman Islands shark populations through an innovative citizen science program
Source: PLoS One. 2025 May 9;20(5):e0319637. doi: 10.1371/journal.pone.0319637 (PMC12064031; doi:10.1371/journal.pone.0319637)
Supplement: S5 Table — Test statistic (Z) and p-values are reported and significant differences, at the 0.05 level, are marked with * . (PDF) [file pone.0319637.s008.pdf]

**Island**	**Test statistic**	**Cayman Brac**	**Grand Cayman**
		sharks (n = 8 species)	
**Grand Cayman**	Z	-2.623	
	p	**< 0.004***	
**Little Cayman**	Z	-10.378	-11.281
	p	**< 0.001***	**< 0.001***
		nurse shark	
**Grand Cayman**	Z	-3.879	
	p	**< 0.001***	
**Little Cayman**	Z	-12.713	-12.981
	p	**< 0.001***	**< 0.001***
