# Supplementary material for: The Sharklogger Network—monitoring Cayman Islands shark populations through an innovative citizen science program
Source: PLoS One. 2025 May 9;20(5):e0319637. doi: 10.1371/journal.pone.0319637 (PMC12064031; doi:10.1371/journal.pone.0319637)
Supplement: S6 Table — Test statistic (Z) and p-values are reported and significant differences, at the 0.05 level, are marked with * (PDF) [file pone.0319637.s009.pdf]

**Grand Cayman**	**Test statistic**	**E**	**NE**	**NS**	**NW**	**SE**	**SW**
			Caribbean reef shark				
**NE**	Z	16.319					
	p	**< 0.001***					
**NS**	Z	12.013	4.935				
	p	**< 0.001***	**< 0.001***				
**NW**	Z	22.687	5.068	-2.701			
	p	**< 0.001***	**< 0.001***	**0.004***			
**SE**	Z	26.494	7.972	-1.495	2.895		
	p	**< 0.001***	**< 0.001***	0.068	**0.002***		
**SW**	Z	12.059	5.110	0.198	2.917	1.733	
	p	**< 0.001***	**< 0.001***	0.422	**0.002***	0.042	
**W**	Z	37.587	13.869	0.247	8.291	5.071	-0.030
	p	**< 0.001***	**< 0.001***	0.402	**< 0.001***	**< 0.001***	0.488
			nurse shark				
**NE**	Z	-0.291					
	p	0.386					
**NS**	Z	-1.090	-0.948				
	p	0.138	0.172				
**NW**	Z	-13.398	-11.817	-4.342			
	p	**< 0.001***	**< 0.001***	**< 0.001***			
**SE**	Z	-4.052	-3.354	-0.513	9.140		
	p	**< 0.001***	**< 0.001***	0.304	**< 0.001***		
**SW**	Z	-2.308	-2.147	-0.916	3.040	-0.726	
	p	**0.011***	**0.016***	0.180	**0.001***	0.234	
**W**	Z	-4.975	-3.941	-0.542	10.759	-0.044	0.729
	p	**< 0.001***	**< 0.001***	0.294	**< 0.001***	0.483	0.233
			hammerhead spp.				
**NE**	Z	-2.526					
	p	**0.006***					
**NS**	Z	-0.818	0.259				
	p	0.207	0.398				
**NW**	Z	-2.602	0.037	-0.244			
	p	**0.005***	0.485	0.404			
**SE**	Z	2.011	4.250	1.605	4.393		
	p	**0.022***	**< 0.001***	0.054	**< 0.001***		
**SW**	Z	1.138	2.160	1.437	2.160	0.353	
	p	0.128	**0.015***	0.075	**< 0.001***	0.362	
**W**	Z	2.893	5.327	1.796	5.598	0.422	-0.216
	p	**0.002***	**< 0.001***	0.036	**< 0.001***	0.336	0.414
