# Supplementary material for: The Sharklogger Network—monitoring Cayman Islands shark populations through an innovative citizen science program
Source: PLoS One. 2025 May 9;20(5):e0319637. doi: 10.1371/journal.pone.0319637 (PMC12064031; doi:10.1371/journal.pone.0319637)
Supplement: S7 Table — Test statistic (Z) and p-values are reported and significant differences, at the 0.05 level, are marked with * . (PDF) [file pone.0319637.s010.pdf]

Little Cayman	Test statistic	E	N	S	Cayman Brac	E	N	S
				Caribbean reef shark				
**N**	Z	-3.546			**N**	0.000		
	p	**< 0.001***				0.500		
**S**	Z	0.325	1.859		**S**	-6.920	-13.824	
	p	0.373	**0.032***			**< 0.001***	**< 0.001***	
**W**	Z	-0.304	6.082	-0.483	**W**	-1.365	-3.443	15.029
	p	0.381	**< 0.001***	0.314		0.086	**< 0.001***	**< 0.001***
				nurse shark				
**N**	Z	-1.793			**N**			
	p	**0.037***						
**S**	Z	-0.795	-0.096		**S**		N/A	
	p	0.213	0.462					
**W**	Z	0.703	4.849	1.165	**W**			
	p	0.241	**< 0.001***	0.122				
				hammerhead spp.				
**N**	Z	-0.978			**N**			
	p	0.164						
**S**	Z	-4.942	-4.921		**S**		N/A	
	p	**< 0.001***	**< 0.001***					
**W**	Z	0.000	1.851	5.244	**W**			
	p	0.500	**0.032***	**< 0.001***				
