# Supplementary material for: The Sharklogger Network—monitoring Cayman Islands shark populations through an innovative citizen science program
Source: PLoS One. 2025 May 9;20(5):e0319637. doi: 10.1371/journal.pone.0319637 (PMC12064031; doi:10.1371/journal.pone.0319637)
Supplement: S8 Table — Test statistic (Z) and p-values are reported and significant differences, at the 0.05 level, are marked with * . (PDF) [file pone.0319637.s011.pdf]

Month	Test statistic	Apr	Aug	Dec	Feb	Jan	Jul	Jun	Mar	May	Nov	Oct
						Caribbean reef shark						
**Aug**	Z	2.289										
	p	**0.011***										
**Dec**	Z	0.598	-1.642									
	p	0.275	0.050									
**Feb**	Z	0.802	-1.167	0.271								
	p	0.211	0.122	0.393								
**Jan**	Z	1.533	-0.524	0.966	0.614							
	p	0.063	0.300	0.167	0.270							
**Jul**	Z	1.244	-1.177	0.584	0.220	-0.497						
	p	0.107	0.120	0.280	0.413	0.310						
**Jun**	Z	4.061	1.708	3.355	2.645	2.058	3.031					
	p	**< 0.001***	**0.044***	**< 0.001***	**0.004***	**0.02***	**0.001***					
**Mar**	Z	1.091	-1.171	0.477	0.148	-0.535	-0.075	-2.889				
	p	0.138	0.121	0.317	0.441	0.296	0.470	**0.002***				
**May**	Z	4.159	1.876	3.473	2.774	2.205	3.161	0.226	3.021			
	p	**< 0.001***	**0.030***	**< 0.001***	**0.003***	**0.014***	**0.001***	0.411	**0.001***			
**Nov**	Z	0.362	-1.875	-0.230	-0.474	-1.178	-0.831	-3.592	-0.709	-3.703		
	p	0.359	**0.030***	0.409	0.318	0.119	0.203	**< 0.001***	0.239	**< 0.001***		
**Oct**	Z	0.674	-1.487	0.095	-0.179	-0.850	-0.459	-3.129	-0.365	-3.253	0.318	
	p	0.250	0.069	0.462	0.429	0.198	0.323	**< 0.001***	0.358	**< 0.001***	0.375	
**Sept**	Z	1.548	-0.456	0.996	0.650	0.050	0.540	-1.946	0.575	-2.093	1.202	0.882
	p	0.061	0.324	0.160	0.258	0.480	0.295	**0.026***	0.283	**0.018***	0.115	0.189

		nurse shark										
**Aug**	Z	-2.855										
	p	**0.002***										
**Dec**	Z	-0.987	1.805									
	p	0.162	**0.036***									
**Feb**	Z	0.318	2.780	1.170								
	p	0.375	**0.003***	0.121								
**Jan**	Z	2.491	5.081	3.347	1.909							
	p	**0.006***	**< 0.001***	**< 0.001***	**0.028***							
**Jul**	Z	-0.463	2.565	0.592	-0.726	-3.028						
	p	0.322	**0.005***	0.277	0.234	**0.001***						
**Jun**	Z	0.793	3.771	1.802	0.350	-1.862	1.331					
	p	0.214	**< 0.001***	**0.036***	0.363	**0.031***	0.092					
**Mar**	Z	1.227	4.066	2.183	0.746	-1.363	1.760	0.490				
	p	0.110	**< 0.001***	**0.015***	0.228	0.086	**0.039***	0.312				
**May**	Z	-1.074	1.801	-0.057	-1.245	-3.480	-0.673	-1.920	-2.304			
	p	0.141	**0.036***	0.477	0.107	**< 0.001***	0.251	**0.027***	**0.011***			
**Nov**	Z	-2.616	0.160	-1.601	-2.584	-4.821	-2.317	-3.492	-3.797	-1.590		
	p	**0.004***	0.437	0.055	**0.005***	**< 0.001***	**0.010***	**< 0.001***	**< 0.001***	0.056		
**Oct**	Z	-0.279	2.420	0.662	-0.549	-2.635	0.142	-1.036	-1.439	0.735	2.209	
	p	0.390	**0.008***	0.254	0.292	**0.004***	0.444	0.150	0.075	0.231	**0.014***	
**Sept**	Z	-1.097	1.406	-0.207	-1.263	-3.252	-0.746	-1.823	-2.172	-0.162	1.235	-0.802
	p	0.136	0.080	0.418	0.103	**< 0.001***	0.228	**0.034***	**0.015***	0.436	0.108	0.211

		hammerhead spp.										
**Aug**	Z	0.500										
	p	0.309										
**Dec**	Z	-0.619	-1.114									
	p	0.268	0.133									
**Feb**	Z	-1.297	-1.734	-0.740								
	p	0.097	**0.041***	0.230								
**Jan**	Z	0.557	0.109	1.110	1.692							
	p	0.289	0.457	0.134	**0.045***							
**Jul**	Z	0.578	0.052	1.221	1.846	-0.068						
	p	0.282	0.479	0.111	**0.033***	0.473						
**Jun**	Z	-1.380	-1.911	-0.708	0.156	-1.813	-2.077					
	p	0.084	**0.03***	0.240	0.438	**0.035***	**0.019***					
**Mar**	Z	-0.770	-1.271	-0.141	0.622	-1.250	-1.389	0.567				
	p	0.221	0.102	0.444	0.267	0.106	0.082	0.286				
**May**	Z	-3.769	-4.301	-3.070	-1.940	-3.961	-4.590	-2.530	-2.959			
	p	**< 0.001***	**< 0.001***	**0.001***	0.026	**< 0.001***	**< 0.001***	**0.006***	**0.002***			
**Nov**	Z	-0.139	-0.629	0.471	1.155	-0.673	-0.712	1.204	0.616	3.548		
	p	0.445	0.265	0.319	0.124	0.250	0.238	0.114	0.269	**< 0.001***		
**Oct**	Z	0.185	-0.287	0.769	1.406	-0.363	-0.349	1.493	0.913	3.751	0.313	
	p	0.427	0.387	0.221	0.080	0.358	0.364	0.068	0.181	**< 0.001***	0.377	
**Sept**	Z	0.139	-0.300	0.685	1.299	-0.372	-0.357	1.347	0.817	3.451	0.260	-0.032
	p	0.445	0.382	0.247	0.097	0.355	0.361	0.089	0.207	**< 0.001***	0.398	0.487
