# Supplementary material for: The Sharklogger Network—monitoring Cayman Islands shark populations through an innovative citizen science program
Source: PLoS One. 2025 May 9;20(5):e0319637. doi: 10.1371/journal.pone.0319637 (PMC12064031; doi:10.1371/journal.pone.0319637)
Supplement: S9 Table — Test statistic (Z) and p-values are reported and significant differences, at the 0.05 level, are marked with * . (PDF) [file pone.0319637.s012.pdf]

Depth (m)	Test statistic	0	6-10	11-15	16-20	21-25	26-30	31-35	36-40	40 <
						Caribbean reef shark				
**6-10**	Z	1.357								
	p	0.087								
**11-15**	Z	0.721	-1.211							
	p	0.235	0.113							
**16-20**	Z	0.024	-2.988	-1.834						
	p	0.490	**0.001***	**0.033***						
**21-25**	Z	-4.567	-9.959	-9.779	-10.452					
	p	**< 0.001***	**< 0.001***	**< 0.001***	**< 0.001***					
**26-30**	Z	-6.122	-15.106	-16.303	-24.088	-2.451				
	p	**< 0.001***	**< 0.001***	**< 0.001***	**< 0.001***	**0.007***				
**31-35**	Z	-3.150	-8.216	-7.924	-8.643	2.886	7.010			
	p	**< 0.001***	**< 0.001***	**< 0.001***	**< 0.001***	**0.002***	**< 0.001***			
**36-40**	Z	-1.373	-2.073	-1.787	-1.495	0.597	1.118	-0.091		
	p	0.085	**0.019***	0.037	0.068	0.275	0.132	0.464		
**40 <**	Z	-2.973	-3.461	-3.274	-3.085	-1.701	-1.376	-2.160	-1.768	
	p	**0.002***	**< 0.001***	**< 0.001***	**0.001***	0.044	0.084	**0.015***	0.039	
**1-5**	Z	1.240	0.476	0.948	1.460	4.586	5.549	3.588	2.051	3.414
	p	0.108	0.317	0.172	0.072	**< 0.001***	**< 0.001***	**< 0.001***	**0.020***	**< 0.001***
						nurse shark				
**6-10**	Z	11.670								
	p	**< 0.001***								
**11-15**	Z	7.834	-7.519							
	p	**< 0.001***	**< 0.001***							
**16-20**	Z	8.990	-7.686	1.787						
	p	**< 0.001***	**< 0.001***	**0.037***						
**21-25**	Z	8.346	-5.664	1.365	0.169					
	p	**< 0.001***	**< 0.001***	0.086	0.433					
**26-30**	Z	10.805	-3.595	6.198	7.457	3.700				
	p	**< 0.001***	**< 0.001***	**< 0.001***	**< 0.001***	**< 0.001***				

**31-35**	Z	7.424	-8.483	-0.932	-3.105	-2.224	-7.600			
	p	**< 0.001***	**< 0.001***	0.176	**0.001***	**0.013***	**< 0.001***			
**36-40**	Z	1.413	-3.774	-1.937	-2.259	-2.257	-3.063	-1.740		
	p	0.079	**< 0.001***	**0.026***	**0.012***	**0.012***	**0.001***	**0.041***		
**40 <**	Z	2.916	-0.481	0.740	0.546	0.520	0.017	0.872	1.695	
	p	**0.002***	0.315	0.230	0.293	0.301	0.493	0.192	**0.045***	
**1-5**	Z	5.481	-1.836	1.021	0.571	0.498	-0.700	1.334	2.219	-0.286
	p	**< 0.001***	**0.033***	0.154	0.284	0.309	0.242	0.091	**0.013***	0.388
						hammerhead spp.				
**6-10**	Z	1.515								
	p	0.065								
**11-15**	Z	1.758	0.343							
	p	**0.039***	0.366							
**16-20**	Z	1.304	-0.716	-1.375						
	p	0.096	0.237	0.085						
**21-25**	Z	0.278	-2.088	-2.655	-2.099					
	p	0.391	**0.018***	**0.004***	**0.018***					
**26-30**	Z	-0.435	-4.125	-5.391	-6.767	-1.491				
	p	0.332	**< 0.001***	**< 0.001***	**< 0.001***	0.068				
**31-35**	Z	0.975	-1.081	-1.623	-0.728	1.256	3.566			
	p	0.165	0.140	0.052	0.233	0.105	**< 0.001***			
**36-40**	Z	-1.355	-2.125	-2.224	-2.014	-1.567	-1.277	-1.879		
	p	0.088	**0.017***	**0.013***	**0.022***	0.059	0.101	**0.030***		
**40 <**	Z	-2.837	-3.368	-3.434	-3.296	-3.000	-2.815	-3.207	-1.661	
	p	**0.002***	**< 0.001***	**< 0.001***	**< 0.001***	**0.001***	**0.002***	**< 0.001***	**0.048***	
**1-5**	Z	-1.351	-2.598	-2.770	-2.473	-1.741	-1.312	-2.238	0.374	2.099
	p	0.088	**0.005***	**0.003***	**0.007***	**0.041***	0.095	**0.013***	0.354	**0.018***
