# Supplementary material for: The Sharklogger Network—monitoring Cayman Islands shark populations through an innovative citizen science program
Source: PLoS One. 2025 May 9;20(5):e0319637. doi: 10.1371/journal.pone.0319637 (PMC12064031; doi:10.1371/journal.pone.0319637)
Supplement: S10 Table — Test statistic (Z) and p-values are reported and significant differences, at the 0.05 level, are marked with * . (PDF) [file pone.0319637.s013.pdf]

Current strength	Test statistic	0	1	2
**1**	Z	-13.226		
	p	**< 0.001***		
**2**	Z	-4.495	2.697	
	p	**< 0.001***	**0.004***	
**3**	Z	-1.356	1.570	0.385
	p	0.088	0.058	0.350
