# Supplementary material for: The Sharklogger Network—monitoring Cayman Islands shark populations through an innovative citizen science program
Source: PLoS One. 2025 May 9;20(5):e0319637. doi: 10.1371/journal.pone.0319637 (PMC12064031; doi:10.1371/journal.pone.0319637)
Supplement: S11 Table — (PDF) [file pone.0319637.s014.pdf]

Shark ID	Sex	Maturity	MLD (km)	SFI (%)
**C107**	F	M	6.46	27.27
**C137**	F	M	N/A	100
**C143**	F	M	0.71	75
**C2**	M	M	1.76	42.90
**C3**	F	M	0.37	63.20
**C4**	M	M	0.55	41.70
**C5**	M	IM	0.63	50
**C6**	F	M	1.07	50
**C7**	F	M	7.31	28.60
**C8**	F	M	7.59	31.80
**C9**	F	M	1.42	36.40
**N1**	M	M	4.56	17.31
**N10**	M	M	2.42	27.27
**N11**	N/A	NA	1.46	33.30
**N12**	F	IM	6.03	50
**N2**	M	IM	10.77	11.90
**N20**	F	IM	1.11	50
**N21**	N/A	IM	N/A	100
**N22**	N/A	IM	N/A	100
**N23**	N/A	M	1.00	66.67
**N3**	F	IM	1.90	33.33
**N4**	F	IM	0.74	50
**N5**	N/A	IM	0.32	50
**N6**	N/A	IM	N/A	100
**N7**	F	IM	7.64	20
**N8**	N/A	IM	4.70	66.67
**N9**	F	IM	0.12	66.67
