# Supplementary material for: The Sharklogger Network—monitoring Cayman Islands shark populations through an innovative citizen science program
Source: PLoS One. 2025 May 9;20(5):e0319637. doi: 10.1371/journal.pone.0319637 (PMC12064031; doi:10.1371/journal.pone.0319637)
Supplement: S12 Table — (PDF) [file pone.0319637.s015.pdf]

Behaviour	Caribbean reef shark<br>(n = 1522 sightings)	nurse shark<br>(n = 2040 sightings)
**Interactions with divers**	• adjectives used by divers: (very) shy, comfortable, curious, inquisitive, nervous, erratic, skittish<br>• shy: immature > mature; male > female<br>• erratic/nervous: no difference in maturity; males only<br>• curious/inquisitive: mature > immature; female > male<br>• comfortable: no immature; only mature; female = male	• adjectives used by divers: (very) curious, friendly, playful, startled, shy, scared<br>• shy: immature only<br>• startled: any demographic<br>• (very) curious: any demographic<br>• (very) friendly/playful/not afraid: any demographic
**Behaviour pattern**	• sharks passed once, multiple times or stayed for the entire dive<br>• sharks arrived at any time during the dive<br>• shark behaviour change during the dive (from shy at the beginning to more curious later)<br>• sharks followed boat/divers from first to second dive site	• sharks swam away (after being disturbed/approached/startled by divers)<br>• sharks followed/stayed with divers (for the whole dive)<br>• sharks followed divers while lionfish hunting, looked into caves when instructor pointed out lobsters<br>• sharks followed boat/divers from first to second dive site<br>• shark came and lay in sand next to divers
**Intraspecific interactions**	• smaller shark avoiding bigger sharks<br>• sharks of similar size and sex are often seen together, especially mature male sharks.<br>• witness of mating event, mating marks on females, courting behaviour (male follows female shark), very girthy females and "suddenly slimmed" down females, females disappear for a few days from their usual home range/dive site<br>• most reproductive evidence was reported from Grand Cayman (E and NW) and Little Cayman (N), no reports on Cayman Brac.	• smaller shark rested on bottom and was disturbed by a larger shark<br>• multiple (2-5) nurse sharks lay on top of each other (in crevice), pair of nurse sharks swimming together<br>• very girthy female sharks, very small (new-born) sharks<br>• reproductive evidence was reported from all Cayman Islands with most reports made on Little Cayman (N).
**Interspecific interactions**	• grouper spp. hunting together<br>• nurse shark swimming together, lots of interactions between the two<br>• jack spp. one or multiple fish accompanying CRS, also seen cleaning the skin of sharks	• southern stingray attacked by nurse shark<br>• Caribbean reef shark swimming together, lots of interactions between the two<br>• blue tang chased by nurse shark

		• unknown spp. nurse shark wiggling around under a ledge, very interested in what's inside
**Habitat use**	• mean depth of shark encounters was 25.52 m ± 0.16 (SE); range = 1.5 - 48.6 m	• mean depth of shark encounter was 11.25 m ± 0.4; range = 0.9 - 37.5 m
	• often sharks come up from the deep to check out divers	• 2/3 of reports: sharks were resting; 1/3 of reports: cruising/swimming sharks; only a small number of sharks were energetic/swimming fast/very active
	• swam along the drop off, often > 10 m below divers (i.e. > 40 m depth)	• only immature sharks were in mangroves and inside sound/ shallows
	• swam on top of reef along the edge of the drop off (approx. 25 – 30 m depth)	• sharks rested often under a ledge/reef/coral head/rock or in a cave, less frequently uncovered on sand
		• sharks swam over reef at mini wall, seldom along the deep wall
		• sharks rolled on sand (removing remoras/cleaning skin)
