# Supplementary material for: The Sharklogger Network—monitoring Cayman Islands shark populations through an innovative citizen science program
Source: PLoS One. 2025 May 9;20(5):e0319637. doi: 10.1371/journal.pone.0319637 (PMC12064031; doi:10.1371/journal.pone.0319637)
Supplement: Attachment — Submitted filename: Response to Reviewers.docx [file pone.0319637.s017.docx]

*PLOS ONE*

Dear Editor,

Thank you for the opportunity to revise our manuscript and for forwarding the comments from the reviewers. In response to the feedback the following amendments have now been made to the manuscript. Please note that the line numbers below refer to the line numbers in the unmarked version of the revised manuscript.

Journal requirements:

1. The manuscript was reviewed, and amendments were made to meet the journal’s style requirements referencing the templates provided (e.g. double-spacing, headings, file names)
2. Additional information were provided in the Methods section to clarify that no permits were required. (line 368-377, also see point #3 below)
3. The full ethics statement was included in the Methods section and additional information is now provided to clarify that no permits were required. (line 368-377)
4. The reference Bewick et al [5] was not found in the manuscript. Please advise what you meant by “Bewick et al. [5]”. In the original manuscript, we did find Reference item #160 Williamson et al. 2020 which met the “unpublished” criteria. This reference was therefore removed in text and from the Reference list.
5. Reference list was amended to reflect the removal of Williamson et al. 2020 because this reference does not meet the journals requirements (see point 4 above). It was not replaced because we did cite others which are sufficient. Refence list was checked against citation in manuscript.

We appreciate the positive feedback from Reviewer #1 about the rigorous protocol and overall manuscript. Thank you for raising concerns and we made changes to clarify each point raised as detailed below:

1. The recording of diver behaviour with regards to lionfish culling was meant to give additional information in case a specific trend was observed (e.g. more shark on specific dives/outliers) and to quantify the dive behaviour of local divers (how much lionfish culling occurred and in what areas). In fact, it was only on a relatively small number of dives that lionfish culling occurred (1,286 dives, 5.6 % of total dives), and these accounted for only a small proportion of shark sightings (338 shark sightings, 7.8 % of total shark sightings). As the reviewer mentioned, this is highlighted in the Results and Discussion to provide the reader with the relevant context to interpret the results.

While it is entirely possible that the lionfish culling might have attracted species which would not normally be in that area, as the reviewer pointed out, our observations show that sharks seemed to be more visible on lionfish culling dives (i.e. come closer to divers and hence into the diver’s field of view) but the species recorded were no different than observed on other occasions. We believe that the difference in ‘visibility of sharks’ might slightly bias the spatial distribution of relative abundance of a species but not influence species composition in an area. We acknowledge the concern and have expanded this section in the Discussion to make it clearer (line 678-683).

2. Thanks for raising this point. Natural migrations/behaviour patterns were not affected as we are referring to movement between adjacent sites <0.3 km apart. Also the sharks do not actually “follow divers”, perhaps a better way of explaining it would be that sharks “reappear at a second, adjacent site (usually about a few hundred meters apart) following movement of the dive boat”. This would happen normally since in most such cases adjacent dive sites would be within an individual shark’s home range. Hence, such observations do not exaggerate the counts and would not classify as “double-counting” because while it is the same shark being seen, it is at two separate sites/dives. The risk of double counting of the same shark at the same site/dive was minimized by taking the number reported by the dive leader after consultation with the dive group (not by adding the counts of separate divers that may all have reported the same shark(s). In addition, various steps were taken during data validation/verification by the coordinator during data cleaning and management (detailed in Data analysis and Discussion). Nevertheless, we appreciate the concern raised by the reviewer and have amended the relevant sections in the Results and Discussion to make the situation clearer (line 590-591, and 851-856). It is also of note that this did not happen on a regular basis but only with a few individual sharks “resident” individuals often known to divers because of their regular occurrence. In short, we don’t believe that such shark behaviour will have impacted the results.
3. Thanks for highlighting the fact that additional references may be useful. Despite other papers on the MPA effect, for sharks in Cayman this seems not to be true. All of our surveys since 2009 show that MPAs don’t harbor greater numbers of sharks than areas outside MPA boundaries. This may be partly because in Cayman original MPAs were designed to “protect areas with high diving pressure” not areas with high biodiversity (briefly mentioned in Introduction line 72-75), hence shark abundance and movements were not taken into consideration when MPAs were established in 1986. In addition, our research has demonstrated that the local sharks regularly move over home ranges greater than the areas covered by MPAs. This is so even though most MPAs in Cayman are believed to have higher prey availability (based on knowledge on currents, waves, other fish abundances and distribution of fishing activities) (mentioned in Discussion starting line 825-834, 835-842, 883-890). We added references to previous research where appropriate as well as a few sentences to the Discussion to make the situation clearer (Discussion line 843-844, 891-893).
4. Thanks for appreciating the significance of these observations. Indeed, these firsthand observations are not common, although as an internet search for papers and images will show (links now added to the Discussion for reference), have become much commoner in recent years. One of these I observed myself (courting behaviour) and I was literally holding my breath because I thought I was about to see a mating event in real life! That case apart, all of the reports of mating were verified through personal communication to double-check what exactly the divers observed. Since the completion of this study, there have been many more reports of mating sharks being observed by local divers, always around the same time period and areas. Such observations used to be considered unusual but have become more common globally as well as in Cayman. This is likely because more divers (and more divers that are trained/aware) are diving in more places where sharks occur. We have added a reminder that this data was validated by the lead author to the relevant section in the Results and Discussion to make it clearer (line 596-599, 906-912).
5. We did encourage the use of cameras by participants, and some divers did use cameras to verify shark species, injuries and behaviour. However, most recorders were dive center leaders, and dive centers have safety protocols that do not allow for camera use by dive staff during dives. To minimize potential misidentifications, any identifications (and numbers) which were questionable, or about which divers had concerns, were checked by the coordinator who would validate through personal communication including referencing pictures, if necessary (detailed in Sample Protocol line 83-92, 127-142). Overall, the data fully support previous scientific surveys using cameras and catch-and-release in Cayman (Ormond et al. 2017, Kohler et al. 2022, 2023), therefore it is reasonable to assume that the training was sufficient to enable divers to identify most shark species correctly. Thanks for raising this point; we have amended the section in the Results and Discussion to make the situation clearer (596-598, 699-708, 728-730, 737-744, 787-789).

We likewise thank Reviewer #2 for the positive feedback regarding the benefits of this manuscript for citizen science and shark conservation research. Both suggestions made by Reviewer #2 have been taken into consideration and amendments to the manuscript have been made as follows:

1. We appreciate the suggestion of adding more information about the spatial diving effort of participants across all three islands. We have this information but initially did not include it in the manuscript in order to keep the paper concise. We agree with Reviewer #2 that the diving effort will aid the reader to better understand the results. We have therefore added a detailed map to the Supporting Information and cited it at the beginning of the Results (line 383-384). We have also amended the relevant section in the Discussion to make this point clearer (line 666-667).
2. We thank the reviewer for pointing out the benefit of expanding on this. More details about the study’s limitations have been added where relevant in the Discussion (e.g. line 669-683). Other sections of the Discussion have been amended to include comparisons with other citizen science programs (E.g. 699-708, 737-744, 946-953).

Sincerely,

Dr Johanna Kohler
Cayman Islands Department of Environment
[Johanna.k.kohler@gmail.com](mailto:Johanna.k.kohler@gmail.com), +1 (345) 926-0135
